# Ensemble CNN to Detect Drowsy Driving with In-Vehicle Sensor Data

**DOI:** 10.3390/s21072372

**Published:** 2021-03-29

**Authors:** Yongsu Jeon, Beomjun Kim, Yunju Baek

**Affiliations:** 1 Department of Information Convergence Engineering, Pusan National University, Busan 46241, Korea; ysjeon@pusan.ac.kr; 2 Department of Smart Software, Yonam Institute of Technology, Jinju 52821, Korea; bjkim@yc.ac.kr

**Keywords:** intelligent vehicle, safety system, driver status monitoring, drowsy driving detection, ensemble CNN

## Abstract

Drowsy driving is a major threat to the safety of drivers and road traffic. Accurate and reliable drowsy driving detection technology can reduce accidents caused by drowsy driving. In this study, we present a new method to detect drowsy driving with vehicle sensor data obtained from the steering wheel and pedal pressure. From our empirical study, we categorized drowsy driving into long-duration drowsy driving and short-duration drowsy driving. Furthermore, we propose an ensemble network model composed of convolution neural networks that can detect each type of drowsy driving. Each subnetwork is specialized to detect long- or short-duration drowsy driving using a fusion of features, obtained through time series analysis. To efficiently train the proposed network, we propose an imbalanced data-handling method that adjusts the ratio of normal driving data and drowsy driving data in the dataset by partially removing normal driving data. A dataset comprising 198.3 h of in-vehicle sensor data was acquired through a driving simulation that includes a variety of road environments such as urban environments and highways. The performance of the proposed model was evaluated with a dataset. This study achieved the detection of drowsy driving with an accuracy of up to 94.2%.

## 1. Introduction

In the vehicle industry, technologies for driver safety and convenience such as autonomous vehicles, intelligent vehicles, and advanced driver assistance systems are being actively researched [1,2,3]. Autonomous vehicles has garnered significant attention from society as well.

SAE International has defined a six-level rating under the J3016 standard to measure the level of autonomous vehicle technology. Currently, various studies are being conducted for partial driving automation systems (level 2) and conditional driving automation systems (level 3) [4,5]. In level 2 or level 3 autonomous driving systems, it is essential to implement the takeover request (TOR) method, wherein the system transfers control authority to the driver. A number of studies [6,7] are being conducted for appropriate TOR methods. One major limitation with the TOR method involves predicting the driver’s response time to the request. For this prediction, a driver status monitoring method that monitors drowsy driving or fatigue driving is required.

Drowsy driving is responsible for 130,000 deaths worldwide annually [8]. According to the National Highway Traffic Safety Administration (U.S. Department of Transportation), about 1000 deaths and more than 90,000 injuries each year are caused by drowsy driving [9]. According to the 2020 announcement by the Korea Traffic Accident Analysis System (TAAS), more than 2500 cases of drowsy driving accidents occur every year. Consequently, drowsy driving has been identified as a major cause of traffic accidents that have occurred on highways over the past 10 years [10].

Previous studies conducted to detect drowsy driving employed methods that can be divided into three categories based on the data used for detection. Three types of data are commonly used: image data, biosignal data, and sensor data.

Image data-based methods acquire data by installing cameras inside the vehicle. To detect drowsy driving, information is generated by characteristics such as the percentage of eyelid closure (PERCLOS), eye movement, and face direction through an image processing method [11,12,13,14,15,16,17]. Since image data directly represents the behavior of the driver during drowsy driving, studies employing image data generally display excellent performance. However, this approach is vulnerable to changes in vehicle illumination and camera lens pollution, and the performance is degraded depending on the driver’s attire such as hats and glasses. Since the position and shooting angle of the camera have an important effect on performance, it is necessary to have it installed by a specialist.

Biosignal data-based methods acquire data through wearable devices. Sensors such as electrocardiograms (ECG) [18,19,20], electromyography (EMG) [21,22,23], and electroencephalography (EEG) [24,25,26] are mainly used. Studies that use biosignal data directly represent the body state of the driver, and they also show excellent performance. However, there is a structural limitation in terms of attaching the device to the body and noise is generated when the contact or grounding is poor, which can affect the reliability of the method.

Sensor data-based methods use two types of sensor data: in-vehicle sensor data and external sensor data. The methods can extract statistical [27,28] and time series features [29,30] from the acquired data. They can detect changes in vehicle movement patterns using the extracted features and traditional machine learning techniques such as random forest [31] and support vector machine (SVM) [32]. In general, they have a low detection accuracy and performance changes drastically depending on driving habits or road types. Because they utilize indirect information such as sudden turns and vehicle movement patterns, it is difficult to determine whether such changes occur due to drowsy driving, erratic driving behavior, or even different road types. However, even if the data acquisition method or install location changes, the amount of change in both the in-vehicle sensor and the external sensor is small. In other words, sensor-based systems are relatively easy to install, making it more practical than previous approaches.

For the same reason as mentioned above, the methods that use sensor data require data acquisition from an actual vehicle. However, it is extremely dangerous to acquire sensor data from an actual vehicle during drowsy driving, and as such, previous studies have collected the data via driving simulations. In numerous studies, a limited simulation environment [33] has been used to restrict the road types or to include some special data such as lane changes and lane positions. In addition, in the collected data, the amount of drowsy driving data is low. Therefore, using a dataset comprising data with such characteristics is not recommended as the reliability of the detection technique can be affected [34].

This study analyzed the characteristics of drowsy driving and categorized them into two types: long-duration drowsy driving and short-duration drowsy driving. To effectively deal with the two types of drowsy driving, an ensemble network that consists of subnetworks based on convolution neural network (CNN), specialized for each type, is presented. By performing a time series data analysis, this study discovered and applied meaningful feature sets based on the type of drowsy driving. In addition, an imbalanced data-handling method is proposed to efficiently train the network. We collected driving data from 17 people of various ages, and the performance of the proposed network was verified with the dataset. Using the collected data, the performance of the proposed network was compared with the existing studies and it demonstrated excellent performance.

The main contributions of this study are summarized as follows:

1. A novel method to detect drowsy driving that is based on an ensemble CNN that uses vehicle internal sensor information is presented.
2. An analysis of various angles to find the characteristics of drowsy driving, an analysis of the duration of drowsy driving, and a time series analysis are performed.
3. A performance evaluation is conducted through a driving simulation dataset consisting of general in-vehicle sensor data for a variety of road environments.

The remainder of this paper is structured as follows: Section 2 describes a quantitative analysis of the drowsy driving data ratio and the duration of the drowsy driving. Section 3 describes the preprocessing process, which includes an imbalanced data-handling method and the feature extraction method through a time series analysis for a meaningful feature set as well as the proposed CNN ensemble network. Section 4 presents the simulation and experimental environments and verifies the proposed ensemble network and analysis method through a performance evaluation that uses the collected data. Section 5 discusses the significance and limitations of this paper. The final section presents the conclusion of this study and discusses future work.

## 2. Analysis on the Duration of Drowsy Driving

We collected about 198.3 h of in-vehicle sensor data through driving simulations. A detailed description of the collected dataset is described in Section 4.1. This section describes the quantitative analysis and results performed on the collected dataset.

A quantitative analysis of the ratio between normal driving data and drowsy driving data was performed on the collected dataset. The data for normal driving and drowsy driving were measured based on the data label.

**Figure 1:**
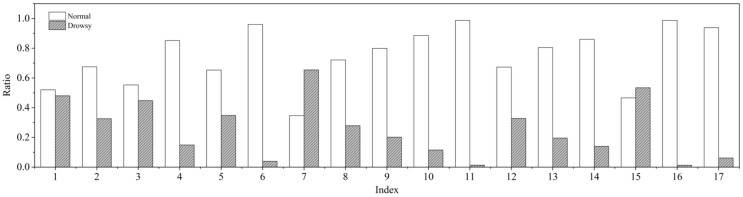
Ratio of normal driving and drowsy driving by driver.

The ratios of normal driving and drowsy driving in relation to the entire data were determined. Figure 1 shows the results from analyzing the data ratio for each driver. According to Figure 1, the ratio differed depending on the driver. For examples, drivers #7 and #15 demonstrated more drowsy driving than normal; however, most drivers demonstrated more normal driving or they were rarely drowsy.

From the analysis results, it was confirmed that the collected dataset was imbalanced, which is approximately 30% of the total data for drowsy driving. The classes in the dataset were balanced with the imbalanced data-handling method that is introduced in Section 3.1.

**Figure 2:**
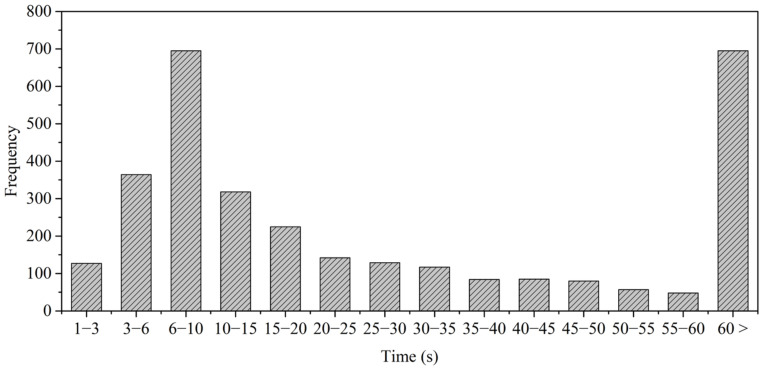
Histogram of the duration of drowsy driving in the dataset.

In addition, a quantitative analysis on the duration of drowsy driving was performed. The duration was calculated from the start of drowsy driving to the end. Figure 2 is a histogram of the drowsiness duration of the entire dataset. This was treated as a single case, considering the data of the duration of being drowsy for more than 60 s to be sparse.

As shown in Figure 2, the two most frequent durations of drowsy driving were between 6 and 10 s and more than 60 s. Inspired by this, we defined a short duration of drowsy driving (SD) and a long duration of drowsy driving (LD) when the durations of drowsy were less than 15 s and longer than 45 s, respectively. We confirmed that SD occurred 1504 times and that LD occurred 880 times during data collection, and the data in both cases accounted for approximately 75.3% of the total drowsy driving.

Analyzing in the time domain, we observed SD for about 3.18 h and LD for about 14.21 h. Despite fewer occurrences of LD, it is observed for a longer time than SD because it is at least 30 s longer in one occurrence.

In addition, we found drivers with a lot of SD and less LD, and vice versa. We divided drivers into two groups based on this characteristic. In the first group, the duration of drowsy driving was distributed around the SD, and there were frequent cases of drowsy driving that briefly occurred during normal driving. However, the other group showed the characteristic in which the duration of drowsy driving was distributed around a LD, and several of them fell asleep slowly and deeply after they started drowsy driving.

## 3. Drowsy Driving Detection Method

**Figure 3:**
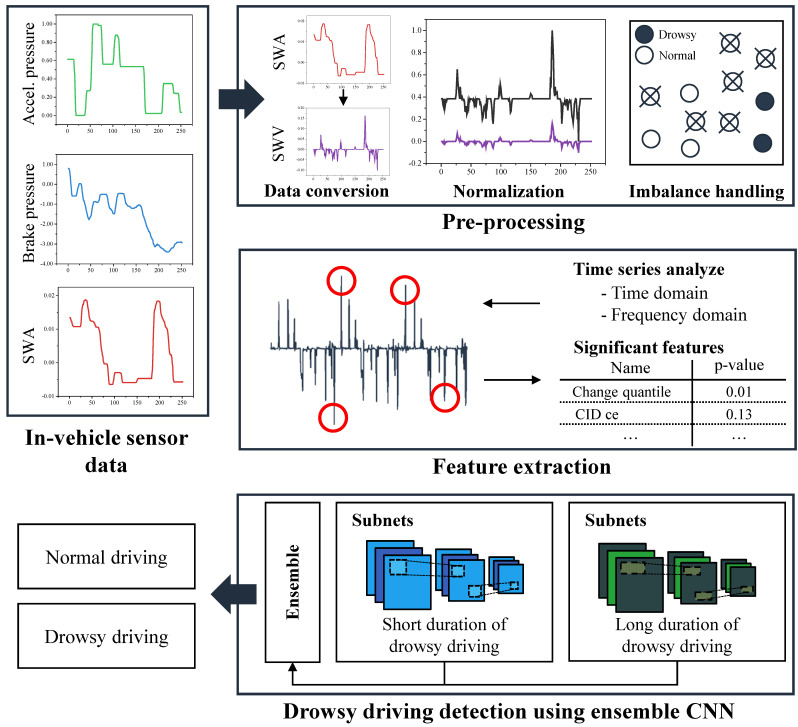
Overall process of the drowsy driving detection method.

The proposed method for detecting drowsy driving comprises the preprocessing step, the feature extraction step, and the drowsy driving detection step. Figure 3 illustrates the overall structure of the proposed method for detecting drowsy driving. Through the preprocessing step, the data collected in Section 2 are converted into segments used for training. Subsequently, the useful features for SD and LD are searched by applying [35], which is a widely known time series analysis method. Based on the widely known time series analysis technique, a feature set specialized for SD and LD is created through useful feature search.

### 3.1. Preprocessing

The preprocessing step consists of the data conversion process, the normalization process, the imbalanced data-handling process, and the segmentation process. The steering wheel velocity (SWV) data were calculated from the steering wheel angle (SWA) data for each lap through the data conversion process. Equation (1) represents the SWV data calculation from the SWA data.

(1) $$SWV_{i}=\frac{SWA_{i}-SWA_{i-1}}{1/SR}\left(i>0\right)$$

where $SWA_{i}$ is the ith SWA value in driving. SR is the data sampling rate. In this study, since the data were acquired at 30 Hz, SR=30 was fixed. SWV data refers to the average change rate of $SWA_{i}$ and $SWA_{i-1}$. The $SWA_{0}$ was initialized to 0.

Following this, the normalization and segmentation processes were performed. In the normalization process, the min–max normalization method was used to obtain the SWA, SWV, accelerator pedal pressure, and brake pedal pressure data.

**Figure 4:**
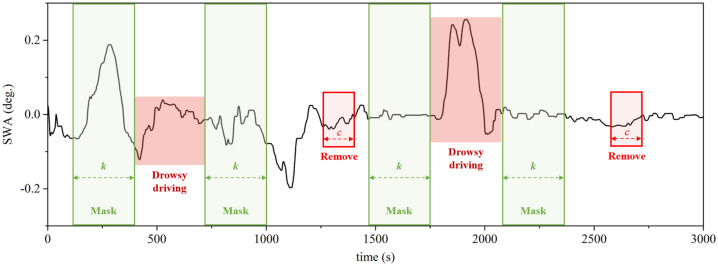
Mask and removal data selected by the proposed imbalanced data-handling method.

Based on the analysis results in Section 2, an undersampling-based imbalanced data-handling method was proposed to resolve the imbalance of the dataset. The proposed method masks the drowsy driving data and the adjacent *k* data in the driving data, as shown in Figure 4. Thus, the temporal features that appear when the state of the driver changes can be protected. Subsequently, random undersampling was performed on the unmasked data. During undersampling, *c* consecutive data were deleted, and from this, the temporal feature of the residual data can be preserved as much as possible. Finally, the imbalance of the dataset was rechecked, and if the imbalance was still severe, undersampling was performed in the same manner.

In the segmentation process, segments of a fixed size were generated through a sliding window method that is commonly used in the time series data. Parameters such as the window size *w* and overlap size *o* are involved in this process. We limited the window size to seconds for low computational complexity. In other words, if the w=60, the segment has 60 s of SWA, SWV, and two pedal pressure data. The overlap ratio can be calculated as o/w, and a high overlap ratio was designed so that many segments can have temporal features that were previously preserved.

### 3.2. Feature Extraction

We used Christ et al. [35] to find features suitable for sensor data from the vehicle through time series analysis. Christ et al. [35] presented an automated process for time series data and provided a technique for calculating *p*-values for various time series features and for selecting the significant ones. In Yadawadkar et al. [34], significant features were extracted for data such as vehicle speed and acceleration using Christ et al. [35].

We calculated *p*-values for a total of 794 time series features for the LD and SD data defined in Section 2. When the calculated *p*-value was less than 0.05, the features were selected as significant features and were used to form the significant feature set for each group. Table 1 and Table 2 list the significant features and *p*-values of LD and SD data.

In Table 1 and Table 2, Brake refers to the brake pedal pressure and Throttle refers to the accelerator pedal pressure. SWA and SWV represent the angle and velocity of the steering wheel, respectively. The features selected as meaningful were 13 features in the LD and 57 features in the SD. More features were selected in SD, estimated to comprise a variety of meaningful features due to the relatively large amount of change in the input data.

We observed the features with high frequency in the selected feature set and analyzed their suitability for the drowsy driving data. First, the change quantile feature was found to be the most selected feature in LD and SD. In LD and SD, it was confirmed that the change quantile was selected as an important feature from the brake pedal pressure data, accelerator pedal pressure data, and SWV data. Change quantile was selected as an important feature in SD of the SWA data. For this reason, we speculate that the change quantile feature contains the most amount of information on drowsy driving. The change quantile is an average of the absolute change in data within the corridor, which sets centering the specific sensor data value. This feature is useful when the data changes severely in a small range and when outliers exist. In the case of in-vehicle sensor data, there are many small changes during normal driving. However, during drowsy driving, the driver usually turns the steering wheel rapidly or steps on the pedal suddenly, and these behaviors appear as outliers.

CID ce [36], Fast Fourier Transform (FFT) coefficient, and aggregation linear trend were commonly selected. CID ce calculates the complexity of time series data utilizing the fact that more peaks and valleys appear as the complexity and variability of the data increases. The CID ce values in LD data were found to have a decreasing trend, and on the contrary, these values showed a tendency to increase in SD data. These trends indicate the sudden steering wheel and pedal operations that are often observed when the condition of the driver changes.

The FFT coefficient is a classic feature that can express time series data and can convert it into frequency components. Naturally, frequent vehicle internal sensor data performed during normal driving appears as high-frequency data. Conversely, when the vehicle is not manipulated during drowsy driving, the data appears in a low-frequency form.

An aggregated linear trend divides segments into smaller chunks and calculates the average value within the chunk. We constructed a linear least squares regression model for representative values of the calculated chunks and then calculated the standard deviation. The value of this feature increases when it is difficult to predict, such as in cases where data change rapidly or irregularly. On the contrary, the value of the feature decreases when it is easy to predict, such as in cases where data change monotonically. In other words, this feature well indicates when it is hard to manipulate the vehicle due to drowsy driving.

### 3.3. Proposed Ensemble CNN

**Figure 5:**
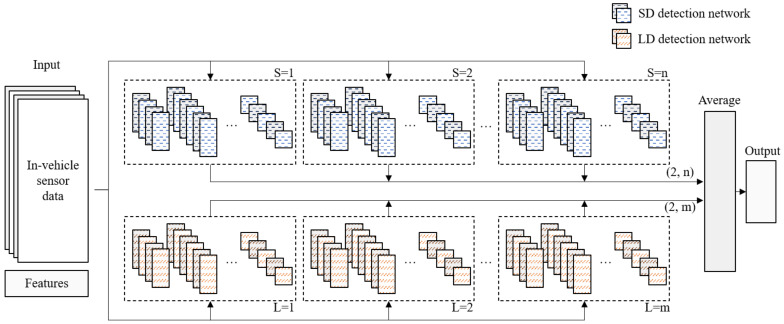
Proposed ensemble convolution neural network (CNN) architecture.

We designed an ensemble network that is composed of CNN-based subnetworks specialized for LD and SD. Figure 5 shows the proposed network structure. The structure and size of the proposed network can be changed by adjusting the number of subnetworks specialized for SD detection (*S*) and the number of subnetworks specialized for LD detection (*L*).

The proposed network utilizes the segment that is output from the preprocessing step and the features that are output from the feature extraction step as inputs. First, the proposed network transforms the segment into a four-channel 2D matrix. The segment contains (SR×w) of SWA, SWV, brake pedal pressure, and accelerator pedal pressure data, where SR is the sampling rate and *w* is the window size. Our proposed network transforms it into a matrix that a has (SR×w)×(4) shape.

Through this transform, we tried to extract the convolutional features not only from adjacent data but also from data after a while. The transformed matrix and statistical features are transmitted to each subnetwork to obtain the probability of normal driving and drowsy driving, respectively. After that, the results are synthesized through the arithmetic mean to determine whether the input data is drowsy.

**Figure 6:**
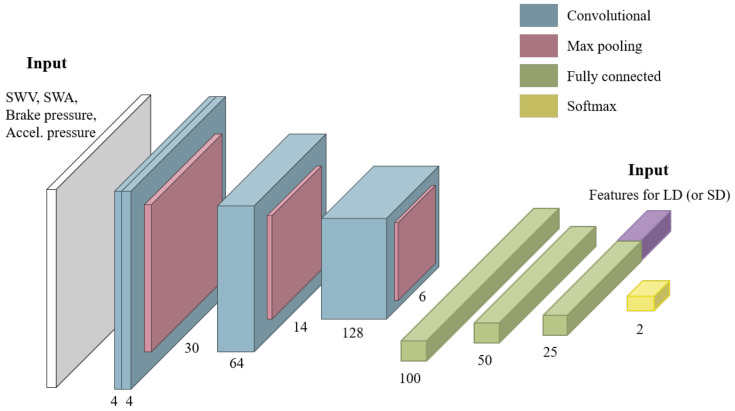
Proposed subnetwork architecture.

The CNN-based subnetwork contains three main features of VGG16 [37]. Figure 6 shows the structure of the subnetwork and the stacked convolution layer in the first block. The convolution-pooling layer structure was applied in the first, second, and third blocks, and it can be observed that the depth gradually increases. Through these structural advantages, the semantic features were effectively extracted from the vehicle sensor data. The statistical features and semantic features were fused in the last fully connected layer. The features that are listed in Table 1 were fused to the subnetwork for the LD detection, and the features listed in Table 2 were fused to the subnetwork for the SD detection. In feature fusion, a simple concatenation operation was used between the semantic and statistical features.

To design a network with good performance, it is necessary to lower the reducible error.

Reducible errors consist of bias and variance, which generally exist in a tradeoff relationship. A high bias means that all the information in the dataset were not considered. Conversely, a high variance refers to a state in which generality is lost due to excessive consideration of dataset information.

The proposed network attempts to reduce bias through a sufficiently deep subnetwork and to reduce variance through a bagging-based technique for each subnetwork. The traditional bagging technique randomly samples training data and divides it to create different subnetworks. In this paper, data from the LD group and SD group, which have different tendencies, were first divided to suppress correlations between subnetworks and to greatly reduce the variance.

Then, *m* different networks for LD detection and *n* different networks for SD detection were pretrained using data of LD group and SD group, respectively. Through this pretrain process, a network group that is specialized for SD and LD detection was acquired.

Subsequently, the proposed ensemble network was trained on the entire training dataset. This manner of learning may cause overfitting concerns; however, the data characteristics of LD and SD are different, and overfitting was suppressed by using enough data.

## 4. Evaluation

In this section, we describe the experimental environment and various experiment results for the performance evaluation of the proposed method. We determine the optimized value of the overlap parameter described in Section 3.1. We evaluate the impact of the proposed imbalanced data-handling method. We also perform an ablation study on the extracted features during the feature extraction process. Finally, we evaluate the performance according to the structure of the proposed ensemble network and compare the performance with exiting studies.

### 4.1. Driving Simulation Environment

We developed a PC-based driving simulator to acquire driving data. We installed the Logitech G920 steering wheel to drive the vehicle in the simulation. The Euro Truck Simulator 2 (ETS2) that was used in the simulator includes a wide variety of roads in Europe, which were closely simulated.

**Figure 7:**
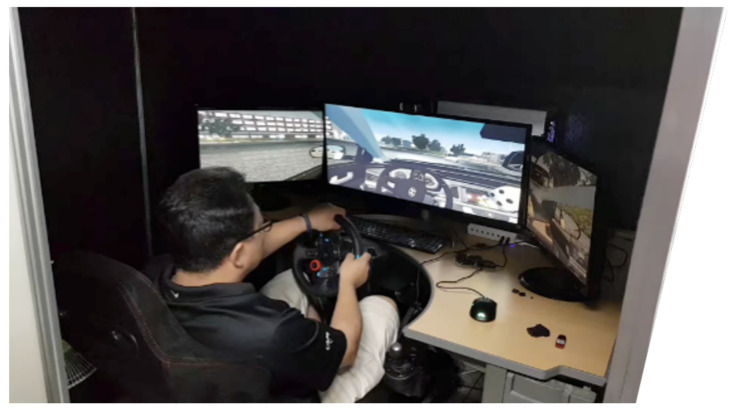
A participant driving in the driving simulator.

We used a vehicle developed using an extension interface called modification (MOD) for data collection. We referred to the specifications of a Hyundai Elantra model: size, acceleration, maximum speed, and rotation radius. Figure 7 shows a participant collecting data in the developed simulator.

**Figure 8:**
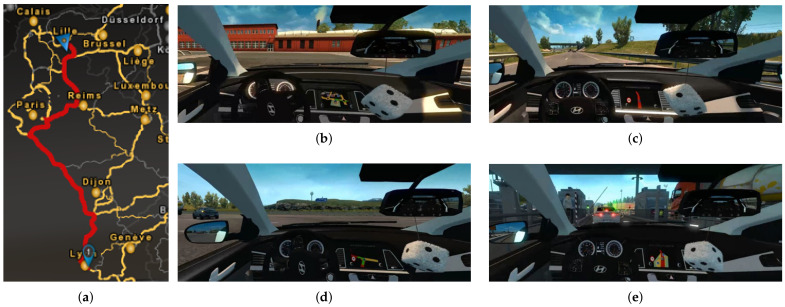
Driving route and road types included in simulation: (**a**) driving route for data acquisition, (**b**) urban road, (**c**) highway, (**d**) crossroad, and (**e**) toll gates.

Additionally, we designed the route using the roads included in the software. The driving route for the data acquisition was set up as shown in Figure 8a. It included urban roads and highways, as shown in Figure 8b,c. In addition, it comprised linear driving and curved driving, as shown in Figure 8d. Road structures such as toll gates, traffic lights, and interchanges, which are shown in Figure 8e, were included. Sensor data such as the SWA, accelerator pedal pressure, and brake pedal pressure during driving were acquired at a sampling rate of 30 Hz. The driver’s face was simultaneously recorded for the ground truth.

To induce drowsy driving when collecting data, we performed simulations in a dark environment where light was blocked between 2 a.m. and 4 a.m. In addition, we advised data collection participants to limit sleep-disrupting behaviors such as naps and caffeine intake before data collection. Preliminary data collection was conducted for 54 participants (21 men and 33 women), who were in their 20 s and 30 s, with more than 1 year of driving experience. Participants who collected inappropriate data due to events such as deviation, speeding, and forward collision; those who were not sleepy at all; and those with mild motion sickness were excluded. As a result, 17 participants (11 males and 6 females) were selected and their data were collected. The driving route was repeatedly driven 20 times, and a total of 198.3 h of simulation data were collected.

Three experts labeled the drowsy state (in seconds) based on the facial movements and expressions of the driver [38,39] in the recorded video. The detailed criteria that were used for labeling are shown in Table 3.

### 4.2. Experimental Environment

We performed the performance evaluation on a PC with an Intel i7-7700 CPU and two Geforce GTX 2080 Ti installed. Additionally, we used the Tensorflow r1.15 and Keras packages in Ubuntu 16.04 environment to implement the proposed ensemble CNN. The application programming interface (API) of the Keras package was utilized for training, validating, and testing the models used in the experiments.

To evaluate the performance of the proposed method in detecting drowsy driving, we ran a hold-out cross validation study. The collected dataset were randomly separated into training, validation, and testing datasets with a ratio of 6:2:2. In other words, we separated the collected dataset across all participants into three subsets.

The performance of the proposed network was evaluated in terms of accuracy, precision, recall, and F1-score. The accuracy was defined as (TP+TN)/(TP+TN+FP+FN), and the precision was defined as TP/(TP+FP). The recall was TP/(TP+FN), and the F1-score was calculated as the harmonic average of the accuracy and recall.

The definitions of TP, TN, FP, and TN are as follows:

- True Positive (TP): Actual state is drowsy driving and inferred as drowsy driving.
- True Negative (TN): Actual state is normal driving and inferred as normal driving.
- False Positive (FP): Actual state is normal driving but inferred as drowsy driving.
- False Negative (FN): Actual state is drowsy driving but inferred as normal driving.

Accuracy refers to the percentage of correct detection for the drowsy driving case and normal driving case and implies the accuracy of the network production. Precision represents the ratio of drowsy driving for the cases that were detected as drowsy driving. That is, if the precision is high, the reliability of the detection result is high. Recall refers to the rate that is detected by drowsy driving among the drowsy driving cases. If the recall is high, the network can detect drowsy driving successfully. In general, recall and precision have an inverse relationship, and precision and recall can be simultaneously considered through the F1-score. The parameters used in the experiment are shown in Table 4.

### 4.3. Segmentation Parameter Optimization

**Figure 9:**
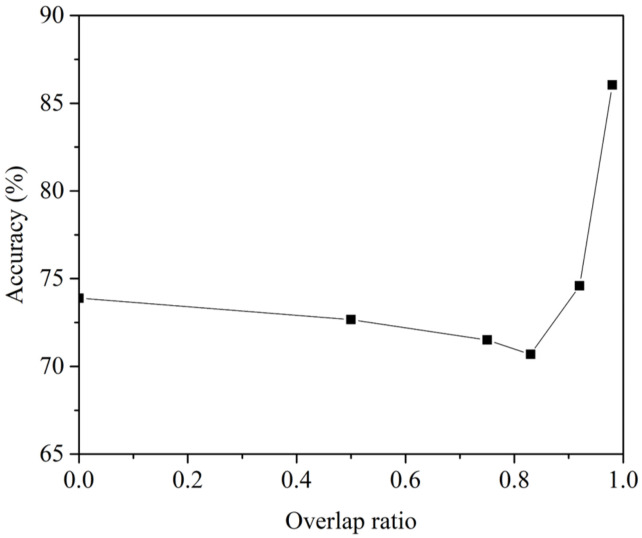
Accuracy of subnetwork according to overlap ratio (w=60).

The overlap parameter is a very important parameter in dealing with sequential data, and we conducted an experiment to find an appropriate value of the overlap parameter *o*. Figure 9 shows the change in accuracy according to the overlap ratio calculated by o/w mentioned above using the proposed subnetwork. Until the overlap ratio reached about 84%, the accuracy of the learned network tended to decrease as the overlap ratio increased. However, it was confirmed that the performance increased sharply from about 84% or more, and the best performance was shown when o=59. In the subsequent experiment, o=59 was set.

### 4.4. Applying the Imbalanced Data-Handling Method

First, we confirmed that, when the imbalanced data-handling method was applied, the percentage of normal driving in the entire dataset decreased from 70.46% to 53.27%. It can be observed that the proposed imbalanced data-handling method effectively controls the ratio of normal driving and drowsy driving in the dataset.

To evaluate the impact of the proposed imbalanced data-handling method, we created two datasets: a balanced dataset and an imbalanced dataset. The imbalanced dataset is the original dataset collected during driving simulation. The balanced dataset is the results from applying the proposed imbalanced data-handling method on the original dataset.

We evaluated the impact of the proposed imbalanced data-handling method by comparing the performance of two networks trained with each dataset, and the results are shown in Table 5. Regarding the network architecture parameters defined in Section 3.3, L=4 and S=4 were used.

The accuracies of the network trained with the balanced dataset and of the network trained with the imbalanced dataset were 93.38% and 94.10%, respectively. However, we confirmed that the recalls of the network trained with the balanced dataset and of the network trained with the imbalanced dataset were 92.79% and 88.40%, respectively.

This implies that the network trained with the imbalanced dataset can determine the normal driving data well but cannot determine the drowsy driving data correctly. A high recall is critical for driver safety due to the characteristics of the detection application for drowsy driving. Therefore, it is necessary to train the network with the balanced dataset that applies the proposed imbalanced data-handling method.

### 4.5. Ablation Study on Extracted Feature Set

Next, an ablation study was performed on the extracted significant feature set. We removed the features with *p*-values closer to the threshold and left *n* features. We defined these *n* features as top *n* features. When measuring the performance, L=4 and S=4 were used as the network architecture parameters, and Table 6 shows the results of the experiment.

An accuracy of 87.69% was achieved when 58 features were used, which exclude the change quantile feature when considering the extracted features. When the extracted features used the top 58 features that include the change quantile, the accuracy was 91.55%. From this, it can be observed that the change quantile is a feature that expresses a relatively large amount of information.

When the top 34 features were used, an accuracy of 84.24% and a F1-score of 82.40% were recorded. When the features were not used, the accuracy was 82.59% and the F1-score was 82.47%. This study confirmed that the accuracy and F1-score decreased as the number of used features decreased.

### 4.6. Experiment Result for the Proposed Ensemble CNN

The performance was compared according to the number of subnetworks in the proposed network. Table 7 and Table 8 show the normalized confusion matrix according to the proposed network architecture. The network with L=1 and S=1 showed the worst performance, with an 86.90% accuracy and an 87.07% F1-score. In the case of L=2 and S=2, the accuracy was 90.81% and the F1-score was 90.67%. When L=4 and S=4, the accuracy was 93.38% and the F1-score was 93.34%. As the number of subnetworks that were included in the network increased, the accuracy and F1-score tended to increase.

This trend was maintained even as the number of subnetworks further increased. In the case of L=6 and S=6, the accuracy was 93.97%, and the F1-score was 93.95%. In the case of L=8 and S=8, the accuracy was 94.07%, and the F1-score was 94.05%. When L=10 and S=10, the best performance was achieved with an accuracy of 94.20% and a F1-score of 94.18%. However, we confirmed that, after L=6 and S=6, the rate of increase in the performance decreased, and it did not increase significantly even when it became larger than L=10 and S=10.

As the number of subnetworks increases, we observed that both training time and inference time were increased. When L=6 and S=6, it took about 18.6 h to train the model, and when L=10 and S=10, it took about 37.8 h. Similarly, when L=6 and S=6, the inference time excluding feature calculation is 228 ms, and when L=10 and S=10, it took about 459 ms.

Table 8 is a normalized confusion matrix showing the performance when the model structure parameter is asymmetric. Previously, it was demonstrated that performance increases as the number of subnetworks increases even when the number of subnetworks is asymmetric, similar to when the number of subnetworks specialized for LD detection and subnetworks specialized for SD detection increase to the same number. When L=1 and S=2, S=4, and S=8, accuracies of 89.59%, 91.04%, and 91.76% were recorded, respectively. Conversely, when S=1 was fixed and L=2, L=4, and L=8, 88.28%, 91.81%, and 92.15% were recorded, respectively.

We confirmed that, when *L* = 2 and *S* = 6, the accuracy was 92.36% and the F1-score was 92.35%. In the case of *L* = 3 and *S* = 5, the accuracy was 92.92% and the F1-score was 92.83%. If the value of the parameter is reversed, the performance changes slightly. When *L* = 6 and *S* = 2, the accuracy was 92.69% and the F1-score was 92.71%. When *L* = 5 and *S* = 3, the accuracy was 92.71% and the F1-score was 92.95%.

**Figure 10:**
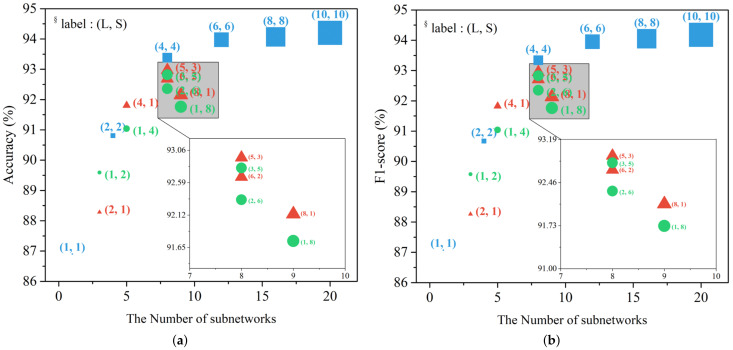
Performance comparison according to model structure parameter changes: (**a**) accuracy and (**b**) F1-score.

Figure 10 is a graph that summarizes the changes in accuracy and F1-score according to the changes in the model structure parameters *L* and *S* mentioned above. In each graph, the size of the dot represents the size of the model and the label represents the parameter value. A square dot means a case where L=S, and a triangle point means a case where L>S. Finally, the circular dot represents the case where L<S.

When the number of subnetworks in the model structure parameter is less than 8, the performance increases rapidly as the number of subnetworks increases. In the case of L=S, it was confirmed that the range of performance increase was greater than in the case of asymmetrical number of subnetworks.

On the contrary, when the number of subnetworks is 8 or more, the performance change is not large as the number of subnetworks increases, but it can be assumed that the variance in the network is sufficiently reduced through the ensemble technique.

Even when the number of subnetworks is asymmetric, it has been shown that the performance increases as the number of subnetworks increases. It was confirmed that the case of L>S has a better performance than the opposite case. According to the analysis results in Section 2, the collected dataset has more LD data than SD data, and we segmented the same window size (*w*). Therefore, it is estimated that it is more advantageous to detect LD better than SD.

Finally, the performances of the traditional machine learning techniques, other deep learning networks, and the previous studies were compared. Table 9 shows the results of the comparison between the machine learning techniques and the other networks. We used L=10,S=10 as the architecture parameters of the proposed network. The deep neural network (DNN) for the performance comparison used a network in which the fully connected (FC) layer was overlapped five times. The output sizes of each layer were set as 128, 256, 256, 64, and 2. We set the activation function of the last layer to a softmax function for the detection result.

One-dimensional CNN consists of two 1D convolution layers and the two FC layers. The parameters of the convolution layers used in 1D-CNN are as follows: the number of filters is 64 and 128, the kernel size is 2, and the stride is 1. The output sizes of two FC layers are 64 and 2, and the activation function of the last layer used the softmax function. We designed the long short-term memory (LSTM) network to have a structure of 3 consecutive LSTM layers and 2 FC layers. Each LSTM layer had 300 units, and the output sizes of the FC layer were set to 64 and 2. In the case of the random forest model, which is a machine learning technique, 100 estimators were used.

As a result of the performance comparison, it was shown that the proposed network outperformed the other networks on all the performance measures. In particular, the LSTM network has an advantageous architecture for analyzing the time-series data such as the sensor data that is used in this study. It shows a higher performance than the DNN, CNN, and random forest models; however, it has a lower performance than the proposed network.

We also performed comparisons with previous studies. Previous studies [27,29,31,40] used traditional machine learning-based methods, whereas Arefnezhad et al. [33] proposed a CNN–LSTM network to detect drowsy driving.

As shown in Table 10, SWA data are used in many studies and pedal pressure data are also used to detect drowsy driving [29,31,33,40]. Although not used in this paper, in-vehicle sensor data such as lateral acceleration data [33] and vehicle speed [27] are also used. In addition, deviation of the vehicle position in a lane [27,33,40] or PERCLOS [27] are used to improve the detection performance.

McDonald et al. [31] collected approximately 108 h of data from 72 participants. The data from the collected dataset in McDonald et al. [31] were segmented to a 60 s window and sampled at a reduced frequency of 1 Hz. Zhang et al. [27] collected about 27 h of data from 27 participants on a circular highway track. The collected data in Zhang et al. [27] were segmented into 600 s non-overlapping windows. Arefnezhad et al. [33] collected data through a driving simulation from 13 participants on a monotonous highway at a sampling rate of 100 Hz that was used as input to the network without any preprocessing. Krajewski et al. [40] collected data from 12 participants through simulations for about 11 h and segmented the collected data. Each segment had a length of 240 s. Unlike other studies, Li et al. [29] collected data from real vehicles for about 14 h from 6 participants. The collected data in Krajewski et al. [40] were sampled to 60 s interval.

In Li et al. [29], an approximate entropy feature was created from SWA data, and in Zhang et al. [27], average and standard deviation were calculated from vehicle speed and lateral position. Similarly, we extracted the approximate entropy feature from brake pedal pressure for SD detection.

We also compared the accuracy performance with the reported results of previous studies [27,29,31,40]. Additionally, the network that was proposed in Arefnezhad et al. [33] was implemented, and the performance was evaluated through the collected dataset. According to Table 10, the proposed method showed superior performance compared to other previous studies. It can be observed that the characteristics for the duration of drowsy driving that were used in this study are very effective. For example, in [40], a variety of features were extracted from several domains and used; however, the performance was relatively low, with an accuracy of 86.10%.

In Arefnezhad et al. [33], the achieved accuracy was 95.05%, but when applied to our collected dataset, it was 84.95%. We speculate that these results are due to the various road conditions and implementation constraints included the simulation. As mentioned above, the dataset was collected on a monotonous highway at a sampling rate of 100 Hz. The dataset in our study was collected on more complex road environments at sampling rate of 30 Hz. In the dataset that was collected, the lateral deviation data that was used in Arefnezhad et al. [33] did not exist; thus, only the SWA, SWV, yawn rate, and lateral acceleration data were used.

## 5. Discussion

From the collected dataset, we found that the duration of drowsy driving is distributed around short and long periods of time. Inspired by this, we designed an ensemble CNN composed of subnetworks specialized for detecting short and long durations of drowsy driving. We confirmed that it is effective in detecting drowsy driving.

Previous studies have determined and utilized the unique features of vehicle control patterns to detect drowsy driving [29,31,33]. In this study, the significant features were obtained through time series analysis. Based on a comparative stukdy, we confirmed that it is effective to divide the drowsy driving into two classes according to duration.

We showed that the proposed ensemble CNN works well in the simulation environment with various road types, but significant research is required to apply the same to the real world. For example, an in-vehicle sensor from an actual vehicle is less accurate than a simulator. In other words, since sensor data in a vehicle is noisier than a simulator, further studies with robustness are needed.

The drowsy driving detection method should have a short inference time because the detection method must observe the changing drowsiness state of the driver in real time. In this paper, a large number of significant features and a large neural network model are used; thus, high computing power would be required to obtain a short inference time. However, it is difficult to satisfy this in an actual vehicle environment. Considering these points, we plan to study drowsy driving detection methods that can work in real vehicles as future work.

## 6. Conclusions

This study proposes a method to detect drowsy driving using vehicle sensor data that comprise the steering wheel angle, accelerator pedal pressure, and brake pedal pressure data. Drowsy driving was classified into two types, long-duration drowsy driving and short-duration drowsy driving, according to the duration of drowsiness, and an ensemble CNN composed of subnetworks specialized for each drowsy driving detection was implemented. Through a time series analysis, significant features such as change quantile, CID ce, FFT coefficient, and aggregation linear trend were extracted to describe long-duration drowsy driving and short-duration drowsy driving effectively, along with an imbalanced data-handling method. To verify the proposed model, a dataset comprising 198.3 h of in-vehicle sensor data was acquired through a driving simulation involving 17 participants.

The proposed ensemble CNN achieved an accuracy of up to 94.20% and a F1-score of 94.18%. By performing a comparative study, it was demonstrated that the ensemble CNN has excellent performance. In addition, this study confirmed that the extracted features and imbalanced data-handling methods are effective for the proposed network in detecting drowsy driving.

## Figures and Tables

**Table 1:** Significant feature list for long duration of drowsy driving (LD) data.

Type	Name	*p*-Value	Type	Name	*p*-Value
SWV	CID ce	0.004	Brake	Large standard deviation	0.035
SWV	Change quantiles	0.007	Throttle	Value count	0.038
SWV	FFT coefficient	0.017	Throttle	Change quantiles	0.041
Brake	AR coefficient	0.018	Throttle	Change quantiles	0.042
Throttle	CID ce	0.024	Throttle	Pct. of reoccurring data points	0.043
Throttle	Agg linear trend	0.025	Throttle	Pct. of reoccurring values	0.045
Throttle	FFT coefficient	0.034			

**Table 2:** Significant feature list for short duration of drowsy driving (SD) data.

Type	Name	*p*-Value	Type	Name	*p*-Value
Brake	Mean abs change	0.003	SWV	Mean abs change	0.022
Brake	Linear trend	0.004	Brake	Approximate entropy	0.024
Brake	Mean	0.004	Throttle	CID ce	0.025
Brake	Ratio beyond r sigma	0.006	Throttle	Change quantile	0.026
SWV	Kurtosis	0.007	Brake	Change quantile	0.027
Brake	Index mass quantile	0.008	SWV	AR coefficient	0.029
Brake	Longest strike below mean	0.009	Brake	C3 lag	0.029
Throttle	Linear trend	0.011	SWA	AR coefficient	0.031
Brake	Count below mean	0.012	SWV	Change quantiles	0.032
Brake	Longest strike above mean	0.012	SWV	Mean abs change	0.032
Brake	Number cwt peaks	0.012	Brake	Ratio value num. to time series	0.032
SWA	CID ce	0.013	Brake	Absolute sum of changes	0.033
Throttle	Agg linear trend	0.013	SWV	Skewness	0.034
Brake	Pct. of reoccurring data points	0.013	SWV	Ratio beyond r sigma	0.034
Brake	CID ce	0.014	Brake	Large standard deviation	0.034
Brake	Partial autocorrelation	0.014	Brake	Sum values	0.040
SWA	Change quantiles	0.015	SWV	FFT coefficient	0.041
Brake	Autocorrelation	0.015	Brake	Augmented dickey fuller	0.041
Throttle	FFT coefficient	0.016	Brake	Count above mean	0.041
SWV	Standard deviation	0.017	Brake	Sum of reoccurring data points	0.041
SWV	Variance	0.017	Brake	Number crossing	0.042
SWA	Number crossing	0.017	Brake	Abs energy	0.043
Brake	Pct. of reoccurring values	0.017	Brake	First location of maximum	0.043
SWV	Linear trend	0.018	Brake	Standard deviation	0.043
Brake	Symmetry looking	0.018	Brake	Variance	0.043
Brake	FFT aggregated	0.019	Brake	Binned entropy	0.046
Brake	FFT coefficient	0.021	Brake	Spkt welch density	0.046
Brake	Value count	0.021	Brake	Sum of reoccurring values	0.047
SWV	Absolute sum of changes	0.022			

**Table 3:** Criteria for determining drowsy driving according to driver behavior.

State (Value)	Indicators
Normal (0)	Eyes kept open, head in line, rapid eyeball movement, looking around
Drowsy (1)	Face stretching, yawning, sighing, intentional blinking of eyes, swallowing, face moving up and down (head-banging), slowing down of the eye blink, decreased degree of an open eyelid, open eyes consciously, fully closed eyes, slanted head, dozing

**Table 4:** Parameter list and value in experiments.

Parameter	Description	Value
k	Mask size	60
c	Consecutive data length for undersampling	5
w	Window size for segmentation	60
epoch	Training epoch	500
batch	Training batch size	64

**Table 5:** Normalized confusion matrix of networks trained with the balanced dataset and the imbalanced dataset.

		Predicted Class				
Actual class		Balanced dataset			Imbalanced dataset	
		Normal	Drowsy		Normal	Drowsy
Normal		0.940	0.060		0.998	0.002
Drowsy		0.072	0.928		0.116	0.884

**Table 6:** Performance comparison of the ablation study on a significant feature set.

# of Features	Description of the Feature Set	Accuracy (%)	F1-Score (%)
70	All features (long: 13, short: 57)	93.38	93.34
58	All features without Change quantiles	87.69	85.46
58	Long: Top 9 features, Short: Top 49 features	91.55	90.31
34	Long: Top 6 features, Short: Top 28 features	84.24	82.40
0	None	82.59	82.47

**Table 7:** Normalized confusion matrix of the proposed network with a symmetric parameter.

		Predicted Class							
Actual class		(*L* = 1, *S* = 1)			(*L* = 2, *S* = 2)			(*L* = 4, *S* = 4)	
		Normal	Drowsy		Normal	Drowsy		Normal	Drowsy
Normal		0.856	0.144		0.923	0.077		0.940	0.060
Drowsy		0.118	0.882		0.107	0.893		0.072	0.928
Actual class		(*L* = 6, *S* = 6)			(*L* = 8, *S* = 8)			(*L* = 10, *S* = 10)	
		Normal	Drowsy		Normal	Drowsy		Normal	Drowsy
Normal		0.946	0.054		0.945	0.055		0.945	0.055
Drowsy		0.067	0.933		0.064	0.936		0.061	0.939

**Table 8:** Normalized confusion matrix of the proposed network with an asymmetric parameter.

		Predicted Class							
Actual class		(*L* = 1, *S* = 2)			(*L* = 1, *S* = 4)			(*L* = 1, *S* = 8)	
		Normal	Drowsy		Normal	Drowsy		Normal	Drowsy
Normal		0.897	0.103		0.910	0.090		0.918	0.082
Drowsy		0.105	0.895		0.089	0.911		0.082	0.918
Actual class		(*L* = 2, *S* = 1)			(*L* = 4, *S* = 1)			(*L* = 8, *S* = 1)	
		Normal	Drowsy		Normal	Drowsy		Normal	Drowsy
Normal		0.884	0.116		0.916	0.084		0.924	0.082
Drowsy		0.118	0.882		0.079	0.921		0.081	0.919

**Table 9:** Performance comparison with other approaches.

Method	Accuracy (%)	Precision (%)	Recall (%)	F1-Score (%)
Proposed	94.20	93.90	94.47	94.18
DNN	70.47	69.38	73.27	71.27
1D-CNN	72.79	68.02	61.85	64.79
LSTM	77.13	77.14	77.10	77.12
Random forest	72.66	72.77	8.04	14.48

**Table 10:** Performance comparison and used variables with previous studies.

Study	Used Variables	Accuracy (%)
Proposed	SWA, SWV, pedal pressure	94.20
[33] ^§^	SWA, SWV, yawn rate, lateral acceleration	84.95
[40]	SWA, pedal pressure, lane deviation	86.10
[31]	SWA	79.00
[29]	SWA	78.01
[27]	Lateral position, vehicle speed, PERCLOS	63.89

^§^ Using the collected dataset in our study.
